# Economic evaluation of HCV testing approaches in low and middle income countries

**DOI:** 10.1186/s12879-017-2779-9

**Published:** 2017-11-01

**Authors:** Jake R. Morgan, Maria Servidone, Philippa Easterbrook, Benjamin P. Linas

**Affiliations:** 1 Department of Medicine, Section of Infectious Diseases, Boston Medical Center, 801 Massachusetts Avenue, Boston, MA 02118 USA; 2 Department of Epidemiology, Boston University School of Public Health, 725 Albany St., Boston, MA 02118 USA; 3 World Health Organization, Geneva, Switzerland

**Keywords:** Hepatitis C, Testing, Screening, WHO, Cost effectiveness, Economic evaluation

## Abstract

**Background:**

Hepatitis C virus (HCV) infection represents a major public health burden with diverse epidemics worldwide, but at present, only a minority of infected persons have been tested and are aware of their diagnosis. The advent of highly effective direct acting antiviral (DAA) therapy, which is becoming available at increasingly lower costs in low and middle income countries (LMICs), represents a major opportunity to expand access to testing and treatment. However, there is uncertainty as to the optimal testing approaches and who to prioritize for testing. We undertook a narrative review of the cost-effectiveness literature on different testing approaches for chronic hepatitis C infection to inform decision-making and formulation of recommendations in the 2017 World Health Organization (WHO) viral hepatitis testing guidelines.

**Methods:**

We undertook a focused search and narrative review of the literature for cost effectiveness studies of testing approaches in three main groups:- 1) focused testing of specific high-risk groups (defined as those who are part of a population with higher seroprevalence or who have a history of exposure or high-risk behaviours); 2) “birth cohort” testing among easily identified age groups (i.e. specific birth cohorts) known to have a high prevalence of HCV infection; and 3) routine testing in the general population. Articles included were those published in PubMed, written in English and published after 2000.

**Results:**

We identified 26 eligible studies. Twenty-four of them were from Europe (*n* = 14) or the United States (*n* = 10). There was only one study from a LMIC (Egypt) and this evaluated general population testing. Thirteen studies evaluated focused testing among specific groups at high risk for HCV infection, including nine in persons who inject drugs (PWID); five among people in prison, and one among HIV-infected men who have sex with men (MSM). Eight studies evaluated birth cohort testing, and five evaluated testing in the general population. Most studies were based on a one-time testing intervention, but in one study testing was undertaken every 5 years and in another among HIV-infected MSM there was more frequent testing. Comparators were generally either: 1) no testing, 2) the status quo, or 3) multiple different strategies. Overall, we found broad agreement that focused testing of high risk groups such as persons who inject drugs and men who have sex with men was cost-effective, as was birth cohort testing. Key drivers of cost-effectiveness were the prevalence of HCV infection in these groups, efficacy and cost of treatment, stage of disease and linkage to care. The evidence for routine population testing was mixed, and the cost-effectiveness depends largely on the prevalence of HCV.

**Conclusions:**

The evidence base for different HCV testing approaches in LMICs is limited, minimizing the contribution of cost-effectiveness data alone to decision-making and recommendations on testing approaches in the 2017 WHO viral hepatitis testing guidelines. Overall, the guidelines recommended focused testing in high risk-groups, particularly PWID, prisoners, and men who have sex with men; with consideration of two other approaches:- birth cohort testing in those countries with epidemiological evidence of a significant birth cohort effect; and routine access to testing across the general population in those countries with a high HCV seroprevalence above 2% - 5% in the general population. Further implementation research on different testing approaches is needed in order to help guide national policy planning.

## Background

Globally, in 2015, there were an estimated 71 million people living with chronic viraemic HCV infection [1]. While there are major differences in the epidemic patterns between and within countries, the WHO Eastern Mediterranean Region and the European Region have the highest reported HCV prevalence. In the past, treatment of HCV infection was complex, prolonged and associated with high rates of side effects and cost. The advent of high-efficacy, short duration oral direct acting antiviral (DAA) regimens with high cure rates [2] has generated new enthusiasm to test for HCV infection, and link those infected to care, treatment and cure before the onset of the complications of cirrhosis and end stage liver disease. However, at present, there is still low access to and uptake of testing, and only a small proportion of those infected have been diagnosed and treated [1]. In 2016, a new global strategy on viral hepatitis was launched, with a stated goal to eliminate hepatitis C as public health threat, and bold targets for reduction in incidence and mortality by 2030 - a 90% reduction in new chronic infections and a 65% reduction in mortality compared to 2015 levels [3]. This also includes ambitious targets for scale-up of testing and treatment to help achieve these goals.

The current standard of care for HCV diagnosis requires a two-step testing algorithm involving serological testing to identify those exposed to infection followed by nucleic acid testing for HCV RNA to confirm presence of viraemia and need for treatment, which is costly and challenging for LMICs lacking laboratory infrastructure. Although, generic competition has resulted in marked reduction in costs of DAA therapy over the last one to 2 years [1], there is substantial cost heterogeneity across countries that does not always reflect a nation’s gross domestic product (GDP) or ability to pay, and prices remain unaffordable in many countries [1, 4].

One of the many barriers to increased access to testing has been the lack of global guidelines on optimal approaches to who and where to test, as well as on testing strategies as regard which assays to use and how that can then be adapted into national policy according to epidemiological context. In September 2015, the World Health Organization (WHO) convened a global guidelines development group to support the development of first ever guidance for testing for chronic hepatitis B and C [5]. The WHO guidelines process uses the methodology of Grading of Recommendations, Assessment, Development, and Evaluation (GRADE) to rate the strength of recommendations and quality of evidence [6]. Overall, the formulation of recommendations is based on assessment of the quality of the evidence, the balance of benefits and harms, acceptability, resource use, cost-effectiveness and programmatic feasibility.

Evaluating cost-effectiveness requires two outcome measures [7]: 1) the cost of an intervention and 2) the benefits. Costs include the lifetime cost of patients who are exposed to the intervention and are expressed in dollars or any other currency. Benefits are typically measured in life years gained or quality-adjusted life years (QALY) gained. With these two outcomes, cost and benefit, it is possible to calculate an incremental cost-effectiveness ratio (ICER) - a primary outcome of cost-effectiveness research. The ICER is defined as: (Cost of a new intervention – cost of standard care)/ (Benefit of a new intervention – benefit of standard care). Importantly, the ICER is always defined *incrementally* in comparison to another treatment strategy. In general, a cost-effectiveness analysis (CEA) compares the ICER for a specific treatment to a threshold value. Interventions with an ICER greater than a threshold are rejected as not cost-effective. The threshold value is referred to as the “willingness-to-pay” threshold. This reflects the average return in QALY that we could expect if we did not use the available budget to provide a new treatment but instead invested that money into the current healthcare system. Importantly, it is not an ethical judgement of how much we are “willing” to pay to save a life. The willingness to pay threshold therefore differs by country. For example in the United States, typical willingness-to-pay thresholds range from $50,000 to $100,000/QALY gained. WHO has previously defined willingness to pay thresholds as a function of a nation’s per-capita GDP [8].

We undertook a narrative review of published studies on economic evaluation of testing approaches for chronic HCV infection (ie. general population or focused testing) to contribute to formulation of recommendations in the 2017 WHO guidelines on testing for hepatitis B and C. We summarised the evidence from these studies of cost-effectiveness of testing and treatment, identified the key drivers of cost-effectiveness and highlighted major implementation research gaps to inform future guidance.

## Methods

We undertook a focused review of the literature to identify relevant studies that had evaluated the cost-effectiveness of different HCV testing approaches. Formal systematic review and meta-analysis of cost-effectiveness studies was precluded by the dearth of cost-effectiveness analyses conducted in LMICs and because of the heterogeneity of: settings and populations studied, testing approaches used in different clinical and community settings, outcomes measured, and methods used to evaluate cost-effectiveness. Our goal was not to assign a specific incremental cost-effectiveness ratio to any testing approach. Rather, we sought in this narrative review to identify consistent findings on economic value of testing in different population groups to support overall decision making and formulation of recommendations alongside other evidence, including recent systematic reviews of HCV seroprevalence in different populations. There was a similar companion narrative review undertaken of cost-effectiveness studies of different testing approaches for chronic hepatitis B virus (HBV) infection [9].

### Inclusion and exclusion criteria

As for the HBV narrative review [9], studies for inclusion in this review were selected according to the following PICO framework. Population: General adult population or high risk target populations; Intervention: Screening for chronic HCV infection followed by treatment; Comparator: no testing, status quo, or multiple alternative testing strategies; Outcome: Studies reporting both costs and benefits; and Study Type: Economic Evaluations (including CEAs or CBAs). We included studies published in PubMed, written in English and published after 2000.

### Search strategy

The original guidelines development group meeting was held October, 2015, and we updated the search in January 2017. We searched PubMed for articles published between January 2000 and January 2017, using the following search strategy and combinations of search terms: screen, screening, testing, cost-effective, cost-effectiveness, cost-utility, hepatitis C, HCV, low-income, middle-income, Africa, Asia, India, Egypt, global, and developing countries. We also reviewed the bibliography of relevant papers to identify other studies which we may have missed using only search terms. We did not search any databases other than PubMed, nor did we search the grey literature. Upon completion of the literature search, we extracted relevant data using a predefined template that included first author, year of publication, population studied, country of analysis, ICER, and citation.

### Terminology

We employed the following terminology for classification of HCV epidemic profiles and testing approaches

- **Classification of HCV epidemic profiles:** HCV epidemics around the world are heterogeneous but largely represented by mixtures of three main epidemic patterns for which a specific testing approach is appropriate. This framework was used in the formulation of recommendations in the WHO testing guidelines. These three patterns are: (i) infection related to high-risk behaviours – requiring focused or targeted testing in the highest-risk groups; (ii) infection related to past generalized exposures that have since been identified and removed (i.e. “birth cohort epidemic”) – requiring routine testing among specific birth cohorts that are readily identified and that have a high prevalence of HCV infection; and (iii) generalized population epidemic with high prevalence generally related to a widespread, often iatrogenic, exposure – requiring routine testing throughout the entire population.
- **Classification of different testing approaches:** Viral hepatitis testing can be delivered to different populations and in different settings as part of general population testing, and/or a focused testing approach in most affected or high-risk populations, delivered through either health facility- based or community-based testing. For the purpose of the guidelines and this review, we considered and defined three main possible testing approaches for HCV infection [5]:
  - *Focused or targeted testing of specific high-risk groups:* This approach refers to testing of specific populations who are most affected by hepatitis infection, either because they are part of a population with high HCV seroprevalence, or have a high risk of acquisition because of risk behaviours and/or exposures. This includes PWID, people in prisons and other closed settings, MSM and sex workers, HIV-infected persons, partners or family members of infected persons, and health-care workers. It may also involve testing on the basis of clinical suspicion of viral hepatitis (i.e. symptoms, signs or abnormal liver function tests or ultrasound scan).
  - *“Birth cohort” testing:* This approach means routine testing among easily identified age or demographic groups (i.e. specific “birth cohorts”) known to have high HCV prevalence due to past generalized exposures that have since been identified and removed. General one-time testing among this population avoids the need to identify risk behaviours. Most countries have at least some component of a “birth cohort” epidemic profile for HCV infection.
  - *General population testing:* This approach refers to routine testing throughout the entire population without attempting to identify high-risk behaviours or characteristics. It means that all members of the population should have potential access to the testing services.

## Results

We identified 26 eligible articles. The majority – 24 of them were from Europe (*n* = 14) or the United States (*n* = 10). Thirteen studies evaluated focused testing among specific groups at high risk for HCV infection, including nine studies in persons who inject drugs (PWID) [10–18]; five studies among people in prison [18–22], and one among HIV-infected men who have sex with men (MSM) [23]. Eight studies involved birth cohort testing [24–31], and five studies evaluated testing in the general population [31–35]. There was only one study from a LMIC (Egypt) and this considered general population testing [35]. Most studies were based on a one-time testing intervention, but in one study testing was undertaken every 5 years [24] and in another study among HIV-infected MSM there was more frequent testing [23]. Comparators were generally either: 1) no testing [11], 2) the status quo [12, 17], or 3) multiple different strategies [23]. All studies were cost-effectiveness analyses where ICERs were the primary result, and no cost-benefit analyses were included. The majority of the studies were conducted before access to newer DAA curative treatment; 16 studies used modeled interferon treatment, four studies used DAA treatment, five studies examined both types of treatment (either as comparators or combination treatment), and one study examined the cost-effectiveness of case-identification but did not include treatment (Table 1). Table 1 summarizes characteristics and key findings from the 26 included studies.

**Table 1:** Literature included in the report

Author	Year	Report section	Country	Strategies	DAA or interferon	ICER	Citation
Castelnuovo	2006	Persons who inject drugs	UK	Compared focused case-finding to background testing	Interferon	16,514£/QALY	[10]
Selvapatt	2017	Persons who inject drugs	UK	Compared testing and treatment in a drug treatment unit to no testing and no treatment	DAA	1029£/QALY	[11]
Loubiere	2003	Persons who inject drugs	France	Compared testing with EIA to no testing	Interferon	3825€/QALY	[12]
Leal	1999	Persons who inject drugs	UK	Compared testing and treating PWID to no testing and no treatment	Interferon	9300£/QALY	[13]
Stein	2004	Persons who inject drugs	UK	Compared testing and treating PWID to no testing and no treatment	Interferon	28,000£/QALY	[14]
Thompson Coon	2006	Persons who inject drugs	UK	Compared focused case-finding to background testing	Interferon	16,000£/QALY	[15]
Schackman	2015	Persons who inject drugs	US	Compared on-site and off-site HCV testing offer in a substance abuse treatment program to no HCV testing offer	Both	18,300$/QALY	[16]
Cipriano	2012	Persons who inject drugs	US	Compared several different interval testing strategies for HCV and HIV compared to no testing	Interferon	168,600$/QALY	[17]
Martin^a^	2013	Persons who inject drugs	UK	Compared dried blood spot HCV testing to no testing in addiction services	Interferon	14,600£/QALY	[18]
Martin^a^	2013	People in prison	UK	Compared dried blood spot HCV testing to no testing in prison	Interferon	59,400£/QALY	[18]
Sutton	2008	People in prison	UK	Compared testing in prison to testing only in the community	Interferon	54,852£/QALY	[19]
Martin	2016	People in prison	UK	Compared increased case-finding in prisons to background testing	DAA	15,090£/QALY	[20]
He	2016	People in prison	US	Compared risk-based opt-out testing in prison to no testing	DAA	29,234$/QALY	[21]
Sutton	2006	People in prison	UK	Compared four case-finding scenarios on intake to prison to no case finding efforts	N/A	£6388/case identified	[22]
Linas	2012	HIV-infected MSM	US	Compared interval testing to symptom-based testing	Interferon	57,800$/QALY	[23]
Nakamura	2008	Birth cohort	Japan	Compared national testing programs to no testing, stratified by age group	Interferon	848$-4825$/QALY	[24]
Coffin	2012	Birth cohort	US	Compared testing of individuals born between 1945 and 1965 to general population testing	Interferon	5400$/QALY	[25]
McEwan	2013	Birth cohort	US	Compared testing of individuals born between 1945 and 1965 to risk-based testing	Interferon	28,602$/QALY	[26]
McGarry	2012	Birth cohort	US	Compared testing of individuals born between 1946 and 1970 to risk-based testing	Both	37,700$/QALY	[27]
Rein	2012	Birth cohort	US	Compared testing of individuals born between 1945 and 1965 to the standard of care	Both	35,700$/QALY	[28]
Liu	2013	Birth cohort	US	Compared testing of individuals aged 40–64 to no testing	Interferon	65,749$/QALY	[29]
Wong	2015	Birth cohort	Canada	Compared testing and treatment to no testing and no treatment in 45–64 year olds	Both	36,471$/QALY	[30]
Ruggeri^a^	2013	Birth cohort	Italy	Compared testing to no testing, stratified by age	Interferon	1383–10,432€/QALY	[31]
Ruggeri^a^	2013	General population	Italy	Compared testing to no testing	Interferon	5171€/QALY	[31]
Eckman	2013	General population	US	Compared testing and treatment to no testing or treatment	DAA	47,276$/QALY	[32]
Deuffic-Burban	2009	General population	France	Compared different follow-up schedules for detection of occupational HCV infection	Interferon	€2020/QALY	[33]
Miners	2014	General population	UK	Compared elevated testing of migrants to the UK to the current background rate of testing	Interferon	£23,200/QALY	[34]
Kim	2015	General population	Egypt	Compared testing and treatment to no testing or treatment	Both	Cost saving	[35]

^a^Articles appearing twice contained sub-analyses covering multiple populations

### Focused testing of high-risk groups

There were 13 studies of focused testing among specific groups at high risk for HCV infection from the United States, UK, and France. This included nine studies in persons who inject drugs (PWID) [10–18]; five studies among people in prison [18–22], and one among HIV-infected men who have sex with men (MSM) [23]. Overall these studies found that testing in high-risk groups and in settings with a large proportion of high-risk patients, such as PWID, MSM, people in prisons and HIV-infected persons, was cost-effective.

#### Persons who inject drugs

Of the nine studies in persons who inject drugs (PWID) across many geographical regions [10–18], key findings were that focused testing for HCV among PWID (where HCV prevalence was between 40 and 90% in various cohorts), and in venues with a high proportion of PWID, is cost-effective [10–18], even when the studies assumed poor follow-up rates, limited access to therapy [10, 16] and a high risk of re-infection. The ICER for HCV testing among PWID populations ranged from €3825/QALY to $65,900/QALY (Table 1).

Selvapatt et al. [11] compared HCV testing and treatment with direct acting antiviral therapy among PWID in an urban drug treatment unit to no testing or treatment and found testing and treatment to be cost-effective with an ICER of £1029/QALY [11]. The authors found that testing and treatment remained cost effective even when the prevalence of HCV was reduced by 20% or the uptake of HCV testing was reduced by 20%.

Another recent study by Schackman et al. (2015) was set in community-based substance use treatment programmes in the United States and compared the cost effectiveness of: 1) no HCV testing or offer, 2) referral to off-site HCV testing, 3) on-site rapid HCV testing offer, and 4) on-site rapid HCV and HIV testing offer [16]. All strategies assumed availability of DAA treatment. The authors used information from a national randomized trial of HIV testing strategies conducted at 12 substance abuse treatment programs in the United States, and modeled an undetected HCV prevalence of 11% in this population. On-site rapid HCV testing alone was cost-effective with an ICER of $18,300/QALY, but assuming a U.S. willingness to pay threshold of $100,000/QALY gained, the preferred strategy was on-site rapid HCV and HIV testing which had an ICER of $64,000/QALY compared to on-site rapid HCV testing alone.

Cipriano et al. [17] is the only study that found HCV testing may not be cost-effective in opioid treatment programs. They examined the cost effectiveness of HIV and HCV testing among PWID in opioid replacement therapy [16]. The authors compared strategies of screening individuals for HIV, HCV, or both infections, and concluded that compared to no screening, the preferred strategy was HIV testing every 6 months. Addition of HCV testing resulted in an ICER of $168,600/QALY and was not considered cost effective assuming a willingness to pay of $100,000/QALY. However, importantly this study predated the availability of effective HCV DAA treatment.

#### People in prison

There were five studies among people in prison [18–22] from the UK and United States. Two studies found that the ICER was sensitive to successful continuity of care or initiation of treatment after testing [18, 20] and three found that testing and treating in prisons could reduce HCV transmission outside of prison [18, 21, 36].

One UK-based study compared dried blood spot testing in prisons compared to venipuncture sampling and assumed a range of continuity of care after exiting prison. Overall, they found that HCV case detection using dried blood spot testing in prisons was cost-effective, even when the model assumed low rates of HCV treatment initiation [18]. Under the base case assumption of no continuity of care, dried blood spot testing had an ICER of £59,400/QALY compared to venipuncture, and the ICER decreased as continuity of care after prison increased. If continuity of care was greater than 40% in prison, the ICER for dried blood spot sampling fell below £20,000/QALY.

An emerging literature also suggests that treating HCV in prisons could reduce HCV transmission in the community [18, 21, 36]. The authors determined that testing and treating with DAAs in prisons could prevent 5500 to 12,700 new HCV infections from released inmates [21]. They found testing in prison to be cost-effective with an ICER of $29,200/QALY for a 10-year testing programme compared to a 5-year testing programme over a 30-year time horizon. The testing programme remained cost-effective even when the researchers varied cost and time horizon parameters [21].

Another recent study in the DAA era has compared doubling the rate HCV tests followed by DAA treatment to the same approach followed by interferon treatment [20]. It was assumed that 56% of patients tested would be referred for treatment and 2.5% of PWID referred would be treated within 2 months of diagnosis in prison. Doubling testing followed by DAA treatment was cost effective with an ICER of £15,090 compared to the same approach but treating with interferon [20]. Overall, the ICER decreased as the rate of treatment increased and the cost of DAAs decreased.

#### Sex workers

We found no studies evaluating the cost-effectiveness of HCV testing or treatment specifically among commercial sex workers.

#### HIV-infected men who have sex with men

There was only one study among HIV-infected men who have sex with men (MSM) and it predated the availability of DAA therapy [22]. In this study, simulation modelling found that testing using liver function tests in combination with HCV antibody was cost-effective in an HIV-positive MSM population [23], but was dependent on appropriate linkage to and retention in care. The study assumed a prevalence of 9.8% in HIV infected MSM and found that testing was cost-effective with an ICER of $57,800/QALY. Testing remained cost-effective after extensive sensitivity analyses, including around HCV re-infection incidence and cost [23].

### Routine testing among specific birth cohorts that are readily identified and who have a high prevalence of HCV

There were eight studies that involved birth cohort testing [24–31]:- five from the US, two from Europe, and one from Japan. All of them show that “birth cohort testing” is cost-effective when compared to expanded risk-based testing or the status quo [24–31].

In the United States, Coffin et al. [25] compared birth cohort HCV testing in adults born between 1945 and 1965 to both general population and risk-based testing and found it to be cost-effective, with an ICER of $5400/QALY compared to risk-based testing and this dominated general population testing (where dominating implies a lower cost but higher QALY compared to general population testing). A general population HCV prevalence of 1.6% and a birth-cohort prevalence of 3.3% was assumed, and it was found that testing in this group remained cost-effective as long as HCV prevalence was over 0.53% [25].

In the same year, McGarry et al. [27] evaluated the cost effectiveness of birth cohort testing in those born between 1946 and 1970 to risk-based testing in the United States [27]. The authors modeled treatment with either interferon or DAA depending on genotype (with only genotype 1 eligible for DAA treatment). Compared to risk based testing, birth-cohort testing was preferred with an ICER of $37,700/QALY. In sensitivity analyses the authors found that decreased treatment rates or efficacy would increase the ICER.

Evaluating a wider range of strategies, Rein et al. [28] compared birth cohort testing followed by interferon treatment, birth cohort testing followed by DAA treatment, risk-based testing, and no testing in a general United States population [28]. The authors assumed that 91% of patients offered testing would accept it, 90% of those tested would receive their results, and that 41% of those who tested positive would initiate treatment. The preferred strategy was birth cohort testing followed by treatment with a DAA containing regimen with an ICER of $35,700 compared to risk-based screening. The findings were robust to a range of sensitivity analyses.

### Routine general population testing

There were five studies of testing in the general population [31–35], and all but one of these was from a high income country (HIC) with low (<2.5%) prevalence [31, 32]. Only one was from a LMIC –Egypt, which has a high prevalence of disease [35]. All were conducted using interferon-based regimens and not the newer more effective DAA curative treatments. In the study from Egypt, one-time, routine HCV testing followed by treatment with either pegylated interferon (PEG) and ribavirin (RBV) or PEG-RBV plus an HCV protease inhibitor was concluded to be a cost saving over a 40-year time horizon, a stronger conclusion than cost-effectiveness as it implies testing and treating in Egypt costs less and provides more QALYs than no testing [35]. The authors used averaged age specific HCV prevalence rates that ranged from 5 to 39%, and assumed that 20% of those identified would be successfully treated. The results were robust given a number of sensitivity analyses, and secondary analyses concluded that general testing in intermediate HCV prevalence regions (HCV~2.5%) may be cost-effective with an estimated ICER of $35,102/QALY.

An evaluation of routine general population testing incorporating the use of DAA treatment was conducted by Eckman et al. in 2013 [32]. The authors developed a Markov state transition model to examine testing in an ethnically and gender-mixed adult population with no prior knowledge of HCV status and compared testing followed by treatment to no testing. General population testing was found to be cost effective with an ICER of $47,276/QALY compared to no testing [32]. The authors evaluated a range of HCV prevalence in sensitivity analyses and found that testing and treating was cost effective as long as the prevalence exceeded 0.84%, which was substantially lower than the modeled HCV prevalence of 1.3–1.6%.

Miners et al. considered the benefits of general population testing in a UK population [34]. The authors compared a case-finding approach to the status quo approach to testing, taking the perspective of the UK National Health Service. The case-finding approach was cost effective with an ICER of £23,200 compared to status quo background testing.

#### Drivers of cost–effectiveness

There were several key findings on drivers of cost-effectiveness across the 26 studies reviewed.

1. Prevalence of HCV: The prevalence of HCV was an important driver of cost-effectiveness of birth cohort and general population testing. Coffin et al. found that birth cohort HCV testing was not cost-effective in the U.S. if the prevalence of HCV in the birth cohort was <0.52% [25]. Of note, this prevalence threshold is substantially lower than the estimated HCV prevalence among the U.S. birth cohort [37]. The one study from Egypt demonstrated that general population testing was likely cost-effective for other LMICs as long as general population prevalence was >2% [35].
2. Efficacy of treatment: The efficacy of treatment also impacted on cost-effectiveness of testing. Rein et al. found that when they assumed treatment efficacy was 70%, the ICER of birth cohort testing in the U.S. compared to focused risk-based testing only was $39,600/QALY. When efficacy of treatment was 38%, the ICER increased to $337,000/QALY saved [28]. McGarry et al. investigated a narrower efficacy range, but found that when treatment efficacy was assumed to be 15% lower than in the base case, the ICER for testing increased 25% - from $37,720/QALY to $47,168/QALY [27]. Similarly, Ruggeri et al. found that when treatment efficacy was assumed to be 36% (the low-end for interferon and ribavirin) the ICER of general population testing in Italy was £8478/QALY gained, whereas when efficacy was assumed to be 76% (the high-end for interferon and ribavirin) it was only £1998/QALY [31].
3. Quality of life with early stage HCV infection: The cost-effectiveness of HCV testing is sensitive to assumptions about the quality of life with early stage HCV infection. Rein et al. found that the ICER of birth cohort testing in the U.S. nearly tripled when a normal quality of life was assumed, though it remained well below U.S. willingness to pay thresholds [28]. Similarly, Ruggeri et al. found that the ICER of general population testing in Italy ranged from €3926/QALY when they assumed a low quality of life, to €7570/QALY when they assumed a very high quality of life [31]. Again, however, while the relative movement in the ICERs is large, the absolute value of the ICER was still below willingness to pay thresholds.
4. Fibrosis stage and fibrosis progression: Because the greatest gains from HCV cure are among those who have cirrhosis or advanced fibrosis, the average disease stage of the population that is being screened is important to cost-effectiveness. For example, Liu et al. found that when they assumed that 29% of the U.S. birth cohort born 1945–1965 had moderate to advanced liver disease, the ICER of birth cohort testing was $73,000/QALY. In comparison, when they assumed that 58% had moderate to advanced disease, the ICER reduced to $59,200/QALY [29]. Depending on the willingness to pay in a given setting, changes in assumptions about fibrosis stage and fibrosis progression may alter cost-effectiveness conclusions
5. Linkage to care: In lower prevalence settings, linkage rates have a large impact on cost-effectiveness conclusions. For example, Schackman et al. found that when they assumed the prevalence of undiagnosed HCV at a substance use treatment center to be approximately the same as that in the general population in the U.S. (0.5%), and poor linkage to care (5% link after diagnosis), the ICER of rapid HCV testing compared to no testing was >$300,000/QALY. When they assumed an undiagnosed HCV prevalence among substance users was 11%, however, the ICER of testing was <$50,000/QALY even when linkage was poor [16]. A consistent observation across studies that if there is no linkage, testing is not cost-effective. Martin et al. found that when they assumed no linkage between prisons and the community, testing for HCV in prisons was not cost effective assuming U.K. willingness to pay of £50,000/QALY [18].
6. Cost of testing and treatment: None of the reviewed studies found that the cost of testing was a driver of cost-effectiveness. The cost of HCV treatment does impact ICERs, but typically not enough to move them across willingness to pay thresholds. For example, Rein et all found that varying the cost of therapy from 80 to 120% of base case cost for interferon and ribavirin resulted in only a 25% change in the ICER of birth cohort testing compared to focused risk-based testing in the U.S. [28]

## Discussion

This narrative review of 26 studies of cost-effectiveness of different testing approaches found that testing in high-risk groups and in settings with a large proportion of high-risk populations, such as PWID, MSM, people in prisons, and HIV-infected persons, is likely to be cost-effective in all settings. The review also showed that in countries with evidence of a significant birth cohort epidemic profile, it would likely be cost-effective to expand HCV testing beyond high-risk groups and also implement birth cohort testing. Routine testing in the general population was generally not shown to be cost-effective outside specific settings with high general population prevalence. This review also highlighted the significant lack of literature about the cost-effectiveness of different HCV testing approaches in LMICs. Although, these key conclusions represent important guiding principles for testing policy, each country must determine its optimal testing approach based on local epidemiology, healthcare infrastructure and resources. The WHO testing guidelines also include a strategic framework to guide countries’ decision-making on selecting testing approaches, and summarizes the key steps for assessing and improving the selection of hepatitis testing approaches [5]. This includes setting targets, reviewing the effectiveness of existing testing activities and identifying gaps, and then adjusting programme activities.

### Focused risk-based testing

Testing among these high-risk groups was found to be effective or highly cost-effective in all but one that pre-dated the availability of effective DAA treatment [17]. It was also recognized that such focused approaches in high prevalence populations would likely have high yield on case-finding. The WHO guidelines strongly recommended that those populations at the highest risk of acquisition and transmission of HCV - such as PWID, people in prisons and other closed settings, MSM and sex workers should therefore be prioritized for HCV testing. It was recognized that priority or high-risk groups will differ across countries and settings, and although not addressed in this review, this may include persons who have had tattoos, body piercing or scarification, unsafe medical procedures, received blood products in countries where screening of blood is not carried out routinely, as well as partners and close contacts of people with HCV infection. A further consideration to support a focused testing approach is that although ascertaining high-risk behaviours is a very effective way of identifying persons in need of testing, many people are unwilling to admit to stigmatizing and often criminalized behaviours. We found no studies evaluating the cost-effectiveness of HCV testing or treatment specifically among commercial sex workers, but given the overlap between sex worker and PWID risk groups [38], it is likely that testing for HCV among sex workers would also be cost-effective in most settings, although such highly stigmatized groups remain challenging [39]. A key implementation consideration for focused testing was the importance of ensuring adequate linkage to care after diagnosis, and that testing without access to treatment will have limited impact.

### Birth-cohort testing

Birth cohort testing was found to be cost effective in all published studies, although these were all in countries with well-established HCV birth cohort profiles of higher HCV risk in those greater than approximately 50 years. The WHO guidelines made a conditional recommendation that whenever there is an easily identified age group (e.g. all individuals born in a certain time period), with a well described higher HCV seroprevalence due to historical exposures, then countries might consider implementing “birth cohort” testing as part of a toolkit of potential testing approaches. This was in part because studies have shown that this approach will likely be cost–effective, with higher rates of case finding, including those likely to have more advanced disease. A key advantage of this generally one time testing approach is that it avoids the need to identify specific often stigmatized behaviors as the basis for testing and the need to categorize individuals as being “high-risk.” While a “birth cohort” epidemiological pattern is more commonly associated with North America and Europe, many other countries have some component of a “birth cohort” effect in their HCV epidemic [40], and therefore “birth cohort” testing is likely applicable in many settings. However, country decision makers also need to recognise that a significant proportion of the HCV-infected population may not be captured within the “birth cohort” and that this should be systematically examined based on population demographics and seroprevalence data [41].

### General population testing

Predictive modeling and cost-effectiveness data demonstrated that the combination of focused- and birth cohort testing will likely identify many cases of HCV infection and be cost-effective. Routine HCV testing among the general population was less supported by data from the review, unless HCV general population seroprevalence was greater than approximately 2% [35], as was the case in the study from Egypt – the only analysis from a LMIC. In the WHO guidelines, a conditional recommendation was made to consider general population testing as an additional testing approach in intermediate- and high-prevalence settings where the prevalence of HCV is >2%.

The drivers of cost–effectiveness tended to be seroprevalence, fibrosis progression, treatment efficacy, and linkage to care. However, countries should take caution that one-way sensitivity analyses can be difficult to interpret across settings. Notably, the cost-effectiveness of HCV testing was relatively insensitive to costs of screening and treatment.

The most critical limitation of this review was that all but one study was from a high income setting, mainly United States and Europe, and there was very limited data on cost-effectiveness of different HCV testing approaches from LMICs.

Further evaluation and comparisons of different HCV testing approaches are needed (i.e. routine general population, focused risk-based, birth cohort testing) using different service delivery models (community or health-facility-based). This can take the form of comparative trials, or large scale implementation studies in a range of epidemic settings and populations in LMICs. Key outcome measures should include impact (uptake, case detection and linkage to care and treatment); cost and cost–effectiveness (and key drivers of cost–effectiveness), and proportion of HCV-infected individuals missed by a specific testing approach. Further research into the simplification of testing and care, and integration of hepatitis services with other health services (e.g. HIV, TB services) is also needed to guide how impact and cost–effectiveness can be improved.

## Conclusion

Most countries have mixed epidemic types, with some combination of all the three main components of epidemic profile, ie. infection related to high-risk behaviours; infection related to past generalized exposures that have since been identified and removed and generalized population epidemic. Determining the optimal strategic mix of HCV testing approaches to increase the diagnosis rate, and in particular, the approach to testing outside of high-risk risk groups will depend on a country’s unique HCV epidemic and the resources available for HCV testing and treatment. The use of focused testing in high risk populations was strongly recommended, while outside of high risk populations, there was a conditional recommendation to consider “birth cohort” testing where there was clear evidence for such an epidemic profile, and for general population testing only in intermediate- and high-prevalence settings.
